# The lower pT limit of deep hydrocarbon synthesis by CaCO_3_ aqueous reduction

**DOI:** 10.1038/s41598-017-06155-6

**Published:** 2017-07-18

**Authors:** E. Mukhina, A. Kolesnikov, V. Kutcherov

**Affiliations:** 1 KTH Royal Institute of Technology, 114 28 Stockholm, Sweden; 2 Gubkin Russian State University of Oil and Gas, 119991 Moscow, Russia

## Abstract

The deep abiogenic synthesis of hydrocarbons is possible under the conditions of the asthenosphere. We have found that this process can also occur under the mineral and thermobaric conditions of subducting slabs. We have investigated the abiogenic synthesis of hydrocarbon systems at pressures of 2.0–6.6 GPa and temperatures of 250–600 °C. The determined lower thermobaric limit of the reaction at 280–300 °C and 2–3 GPa corresponds to a depth of 70–80 km during cold subduction. The hydrocarbon fluid formed in the slab can migrate upwards through the network of faults and fractures to form petroleum deposits.

## Introduction

The origin of hydrocarbons addresses such topics as petroleum accumulation^1^, global carbon and hydrocarbon cycles, and the origin of life^2^. During the last decades, theoretical and experimental investigations of hydrocarbon systems under high thermobaric conditions were published^3–5^. The experiments and computations focused on the properties and phase transformation of single hydrocarbons^4, 6^ and complex hydrocarbon systems^7–9^ with chemical compositions similar to those of natural hydrocarbon systems. In the abovementioned studies, the abiogenic formation of hydrocarbons was attributed to the high pressure-temperature chemical reactions between the natural donors of carbon (mineral or fluid) and hydrogen (presumably aqueous fluids) in the mantle. In most of these works, these reactions were attributed to high pressures at temperatures of approximately 1000 °C^5^. These thermobaric conditions correspond with the Earth’s asthenosphere conditions. Previous experiments demonstrated that hydrocarbons can be formed from deep rock-fluid interactions under a wide range of temperatures and pressures of 500–1500 °C and 3.0–8.0 GPa^6, 8–11^. The results of these experiments showed that the water-carbonate interaction under reduced mantle conditions is one of the most preferable paths, leading to the deep formation of hydrocarbons^12^.

However, the subducting slab environment could also be considered for hydrocarbon formation due to the enrichment of slab sedimentary rocks with carbonates^13, 14^ and the abundance of particularly calcite-rich rocks in subduction zones^15–18^.

In this study, we have attempted to determine the lowest thermobaric conditions (and the corresponding depth) at which hydrocarbons could be generated in the slab from carbonates and water (the source of which could be any hydroxyl group-containing mineral presented in the investigated geological environment, e.g. serpentine) in the Earth’s interior.

The experiments were carried out in Toroid-type high-pressure chamber. The analysis of the gases produced was performed using gas chromatography from the gas extracting cell. Raman spectroscopy was used to analyze the solid phase products (see Method). The high-pressure equipment was pressure and temperature calibrated (see Supplemental Materials). The design of high-pressure equipment allowed us to measure the pressure and temperature with an accuracy of 0.1 GPa and 10 °C throughout the experiment, respectively. The quantity of the gas produced was only calculated relatively due to the present technical difficulties of the gas extracting cell calibration (see Supplemental Materials).

According to Kutcherov *et al*.^19^, the general pathway for the formation of hydrocarbons:

1 $$n{{\rm{CaCO}}}_{3}+(3n+1){\rm{Fe}}+(2n+1){{\rm{H}}}_{2}{\rm{O}}\to n{\rm{Ca}}{({\rm{OH}})}_{2}+(3n+1){\rm{FeO}}+{{\rm{C}}}_{n}{{\rm{H}}}_{2n+2}$$

To determine the lower thermobaric limit of reaction (1), we conducted 15 experiments in a temperature range of 250 to 750 °C at pressures ranging from 2.0 to 6.6 GPa. Thermobaric conditions were chosen according to the Earth’s interior pressure-temperature profiles^20^. Experimental conditions and product characterizations are shown in Table 1. The description of high and low yields is presented in the Supplemental Materials (Supplementary Note).

**Table 1:** The conditions of the conducted experiments.

Experiment	P, GPa	T, °C	Redox environment	Results of experiments
1	2.0	600	Iron	High yield of hydrocarbons
2	2.6	600	IW buffer	High yield of hydrocarbons
3	3.0	400	Iron	High yield of hydrocarbons
4	4.0	450	Iron	High yield of hydrocarbons
5	6.5	550	IW buffer	High yield of hydrocarbons
6	6.6	400	Iron	High yield of hydrocarbons
7	2.3	300	Iron	Low yield of hydrocarbons
8	2.6	300	Iron	Low yield of hydrocarbons
9	3.0	300	Iron	Low yield of hydrocarbons
10	4.0	280	Iron	Low yield of hydrocarbons
11	6.5	280	Iron	Low yield of hydrocarbons
12	2.3	250	Iron	Hydrocarbons were not detected
13	3.0	250	Iron	Hydrocarbons were not detected
14	4.0	250	Iron	Hydrocarbons were not detected
15	6.6	250	Iron	Hydrocarbons were not detected

All performed experiments were heated in 60 s, exposed for 2 h, and cooled instantly (quenched).

We replaced initial iron with iron(II) oxide in experimental runs #2 and #5, slightly increasing the oxygen fugacity of the system (reaction from Kutcherov *et al*.^19^).

**Figure 1:**
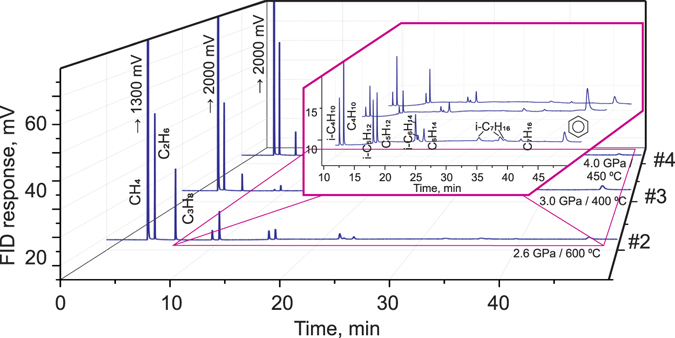
Representative chromatographic spectra of the experiments with relatively high yields of gaseous hydrocarbons.

It was observed that the water-carbonate interaction in the reduced conditions leads to the formation of hydrocarbons in the majority of our experiments. We divided the experiments into two sets: experiments where the formation of hydrocarbons was detected, and experiments in which no hydrocarbons were observed. The results of the first set of experiments show that the hydrocarbon mixture formed contains a prevalence of methane and remains stable at temperatures higher than 250 °C over the entire pressure range investigated (Fig. 1). From the chromatograms shown in Fig. 1, one can see that although methane is the main component of the hydrocarbon system (up to 95%), the mixture contains other saturated hydrocarbons to heptane, their isomers, and even benzene. Although the distribution of hydrocarbons was observed in previous works^8, 19^, this is the first time that the aromatic component, benzene, has been observed as the product of the reaction between calcite and water in the reduced environment. No signs of oxygen-containing hydrocarbons were detected, though, their formation in similar systems was reported in recent works^11, 21^, probably due to either lower temperatures in this research, or the limits of the analysis technique and/or different reaction components, including capsule material.

The results of the second set of experiments (#12, #13, #14 and #15) demonstrate that at a temperature of 250 °C within the pressure range of 2.3–6.6 GPa, hydrocarbons were not formed. These thermobaric conditions lay outside of the reaction zone.

**Figure 2:**
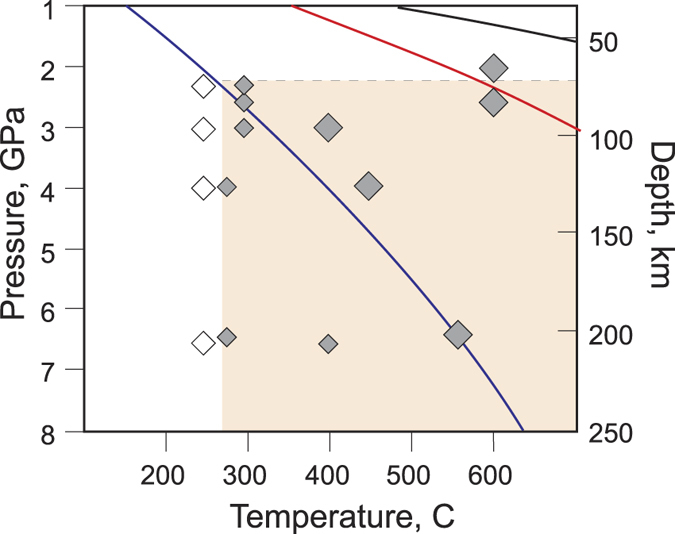
Experiments and geotherms^20^. The ***blue curve*** is a cold slab, the ***red curve*** is a hot slab, and the ***black curve*** is the upper mantle pT profiles. The ***white diamonds*** depict experiments where hydrocarbons are not detected. The ***small gray diamonds*** correspond to experiments with a low hydrocarbon yield. The ***big gray diamonds*** correspond to experiments with a high hydrocarbon yield. The error bars are smaller than the symbol size. The ***pink sector*** indicates the thermobaric zone where hydrocarbon formation is favored.

Figure 2 illustrates the performed experiments along with slab geotherms. In some of the first set of experiments conducted at lower temperatures (#7, #8, #9, #10 and #11), the hydrocarbon yield was significantly low. Combining this result with the results of the second set of experiments suggests that the hydrocarbon formation at pressures from 2.0 to 6.6 GPa occurs at temperature higher than 250 °C. No significant pressure influence was observed. According to the pT-profile of the Earth’s interior^20^, a temperature range of 250–300 °C at 2.0–3.0 GPa corresponds to a depth of 70–80 km of a cold subducting slab. Therefore, the results from our experiments show that the formation of hydrocarbon systems from carbonates and water may occur in the cold slab at a depth of 70–200 km.

The results of the experiments #1 and #2 at the thermobaric conditions corresponding to the conditions in a hot slab show that the formation of complex hydrocarbon systems may also begin at a depth of 70–80 km (Fig. 2).

The solid product formed in experiment #2 was analyzed using Raman spectroscopy (Supplemental Materials, Supplementary Figure 1). The obtained Raman spectra revealed the presence of heavier hydrocarbons in the solid product. Heavy hydrocarbons were observed previously from the system MgCO_3_–Ca(OH)_2_–Fe–SiO_2_ after exposure at 3 GPa and 1400 °C for 24 hours^11^. No free carbon (diamond or graphite) was detected. This result differs from the study of carbonated mafic mineral aqueous transformations, where diamond formation was observed at a pressure of 5.0 GPa and a temperature of 900 °C^22^. This discrepancy is a consequence of the significantly lower temperature used in our experiments compared to that in the experiments conducted by Sverjensky & Huang (2015)^22^.

In Martirosyan *et al*.^23^, the formation of iron carbide was observed as a result of a reaction between iron and magnesite at 6.0 GPa and 1000–1500 °C. In our experiments, iron carbide was not observed. In contrast to the experiments described^23^, we used water as one of the initial components of the reaction.

The observed differences imply that the behavior of the carbonate-iron-water system strongly depends on the thermobaric conditions and could differ in the subducting zone and asthenosphere.

**Figure 3:**
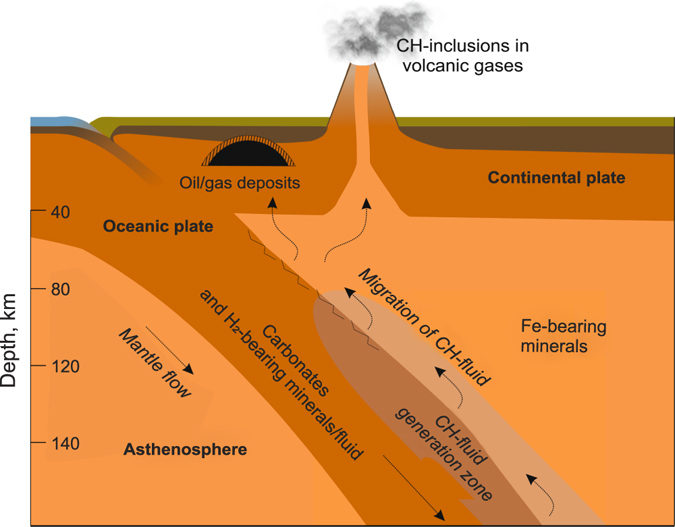
Schematic representation of the generation and migration of a hydrocarbon fluid during subduction.

Fluid migration pathways is an important issue to discuss in the formation of petroleum deposits. Several studies discuss different mechanisms of migration such as hydrocarbon-bearing melt migration within a mantle plume^24^, and volcanic activity^25^. In our opinion, the most favorable path for the formed hydrocarbons to migrate from the subduction zone upwards is by migration along the weakened surface of the slab (Fig. 3).

To conclude, we have demonstrated that the formation of complex hydrocarbon mixtures could occur in the subducting slabs at a depth of 70–80 to 200 km. The formed hydrocarbons can migrate upwards along the weakened surface of the slab to the Earth’s crust and form hydrocarbon deposits. These results may further enlighten the abiogenic origin of hydrocarbons and mechanism of petroleum deposit formation.

## Method

### Experiments in a Toroid-type high-pressure chamber

Pure CaCO_3_ (>99%, Sigma Aldrich), Fe (>99%, Sigma Aldrich), FeO (>99%, Sigma Aldrich), and distillated water were used as starting materials in our investigations. The high pressure-temperature conditions corresponding to those in a subducting slab were modeled using a Toroid-type high-pressure chamber with resistive heating^26^.

CaCO_3_, Fe(FeO) and H_2_O (molar ratio 1:1:1) were mixed and tightly loaded into a steel capsule with a working volume of ~0.5 cm^3^. Stainless steel (Fe~70%) was used as the capsule material to avoid any influence of any additional components on the reaction course since iron was one of the reaction components. The capsule design allowed contact between the mixture and the atmosphere to be avoided. The mixture contained in the sealed capsule was placed in the toroid chamber. Two heaters (graphite/Al_2_O_3_) were placed above and below the capsule, allowing resistive heating to achieve temperatures up to 1800 °C. Reference materials used for pressure (Bi, PbSe, PbTe) and temperature (Ti, Sn, Pb) calibration using the resistance differential (Supplementary Figure 2 in the Supplemental Materials) were placed on the top of the capsule inside the paper disk and covered with aluminum or copper foil for improved conductivity. The toroid chamber with the capsule inside was put between two hard alloy anvils of the high pressure equipment. The assemblage is shown in Supplementary Figure 3 in the Supplemental Materials. The main experimental parameters were indirectly controlled by the “URS-2” press program. The hydraulic pressure and power applied to the anvils was set according to a calibration to create the desired pressure and temperature in the capsule. The program also allowed the rate of heating to the required temperature, the duration of exposure, and the cooling rate to be adjusted. All performed experiments were heated in 60 s, exposed for 2 h, and cooled instantly (quenched).

### Analysis by means of gas chromatography

The analysis of the gaseous products was carried out by means of a gas chromatograph Chromatek-5000 using a gas extracting cell. The cell is an improved version of a previously described modification^19^. The present gas extracting cell (Supplementary Figure 4 in the Supplemental Materials) allows us to send the gas mixture directly from the capsule into the gas chromatograph. After the capsule was taken from the pressure equipment it was placed inside the cell and sealed inside with a rubber ring. After air was taken from the cell by the first blow, a hard alloy needle penetrated the capsule. After penetration, the gas spread out inside the cell and was then sent to the chromatograph for analysis by the second blow.

The specification of the chromatograph Chromatek-5000 was focused on the detection and separation of light hydrocarbons by an Agilent GS-GasPro capillary column in an increasing temperature regime from 60 to 140 °C in 50 min. The column allows to detect even neglectable quantity of the hydrocarbon gas produced. The detection of the separated hydrocarbon components was carried out by the FID detector.

### Analysis by means of Raman spectroscopy

After the analysis of the gaseous product, the solid phase of experiment #2 was extracted from the capsule and analyzed by Raman spectroscopy using a He-Ne laser (wavelength 632.8 nm, power 2 mW) at Bayerisches Geoinstitut. The solid powder was evenly distributed on the surface of a glass plate and examined in a few different areas using the laser with a ~30 µm spot size.

### Data Availability

All data generated or analyzed during this study are included in this published article (and its Supplementary Information files).

## Electronic supplementary material

Supporting Information
