# Supplementary material for: The lower pT limit of deep hydrocarbon synthesis by CaCO3 aqueous reduction
Source: Sci Rep. 2017 Jul 18;7:5749. doi: 10.1038/s41598-017-06155-6 (PMC5515916; doi:10.1038/s41598-017-06155-6)
Supplement: Supplementary file 1 — Supporting Information [file 41598_2017_6155_MOESM1_ESM.doc]

**Supplemental Materials for**

**The lower pT limit of deep hydrocarbon synthesis by CaCO_3_ aqueous reduction**

E. Mukhina^1,2^*, A. Kolesnikov^2^, V. Kutcherov^1,2^

^1^KTH Royal Institute of Technology, 114 28 Stockholm, Sweden (*correspondence: mukhina.e@gubkin.ru)

^2^Gubkin Russian State University of Oil and Gas, 119991 Moscow, Russia

**
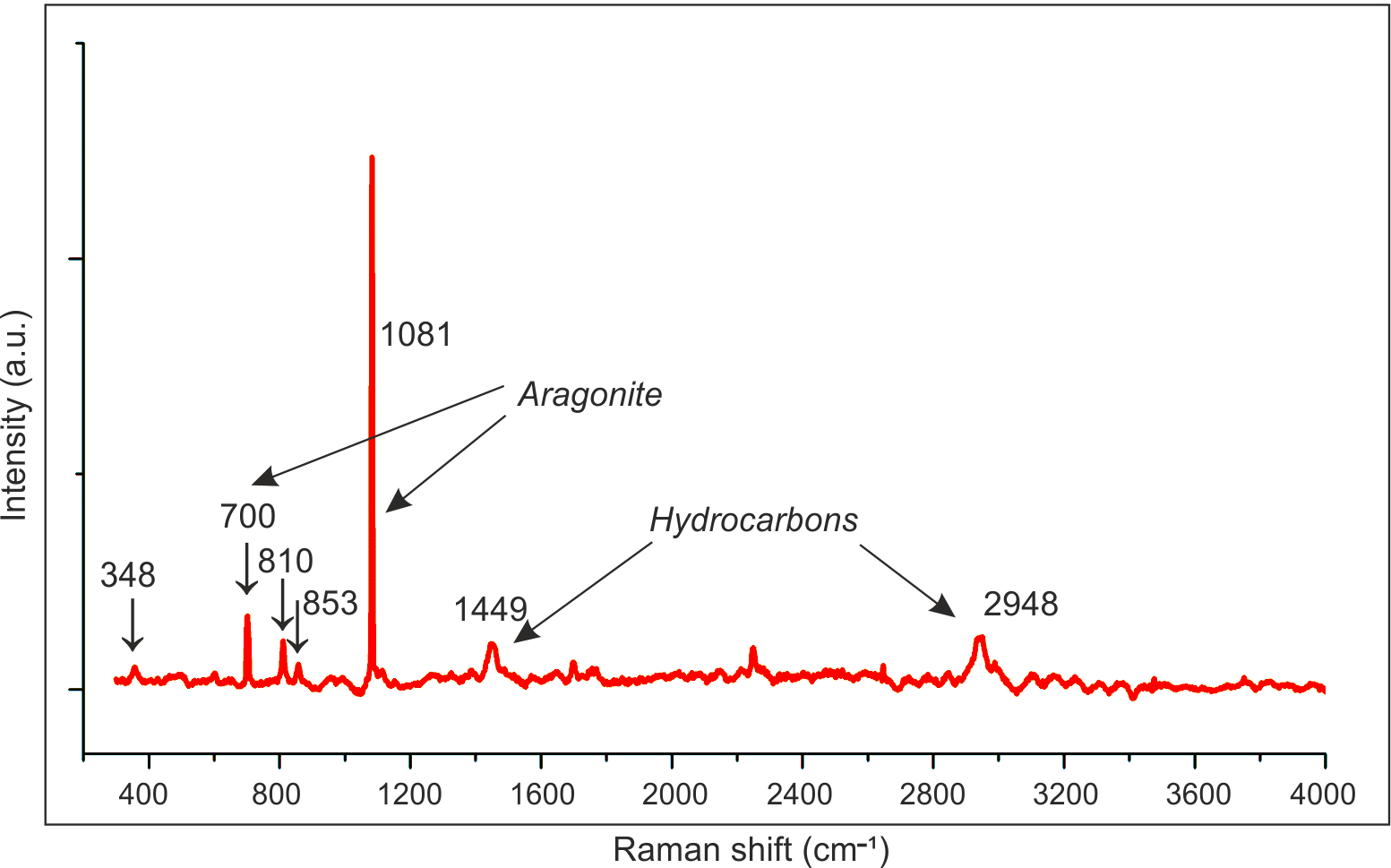
**

**Supplementary Figure 1**. Raman spectra of the solid product formed in experiment #2 (system CaCO_3_-FeO-H_2_O, P = 2.6 GPa, T = 600 °C) taken at ambient temperature and pressure. Peaks at 348, 810 and 853 cm^-1^ may be identified as iron oxides.


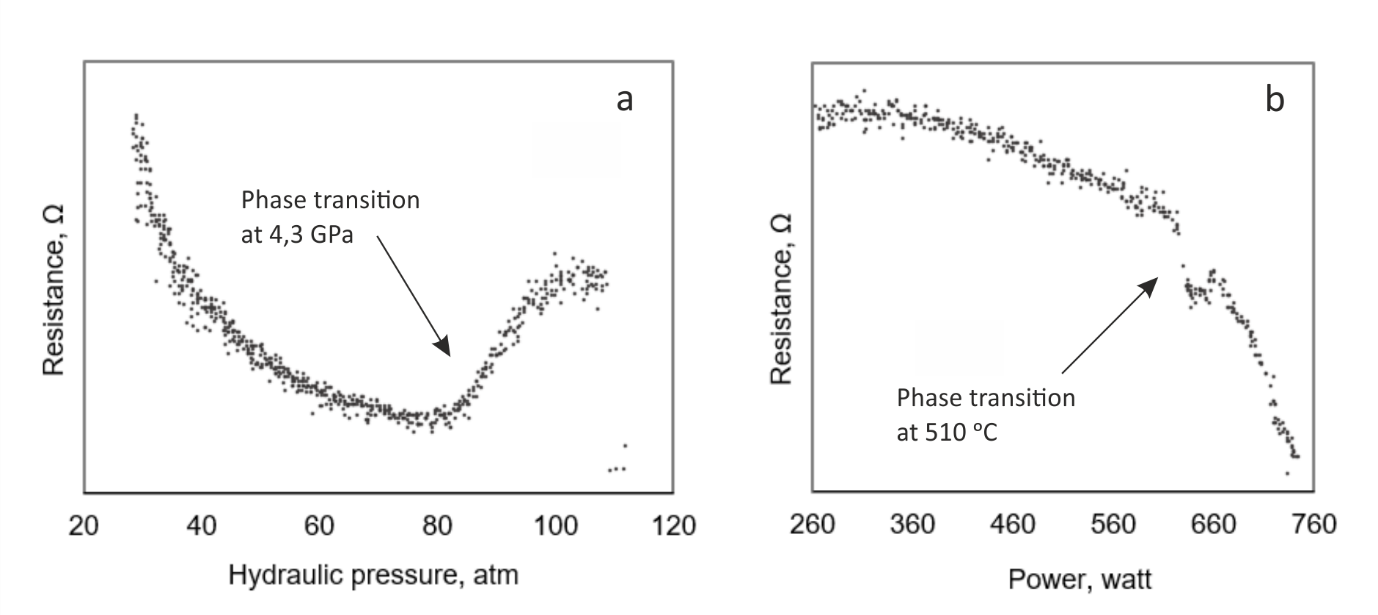


**Supplementary Figure 2**. Representative graphics for Toroid-type high-pressure chamber calibration: a – pressure calibration at ambient temperature, the reference substance is PbSe, the resistance leaps after a phase transition at 81 atm of hydraulic pressure of the equipment, which applies 4,3 GPa to the sample; b – temperature calibration at 2,6 GPa, the reference substance is Pb, the resistance drops after a phase transition at the power of 620 watt of the equipment, which applies 510 °C to the sample.


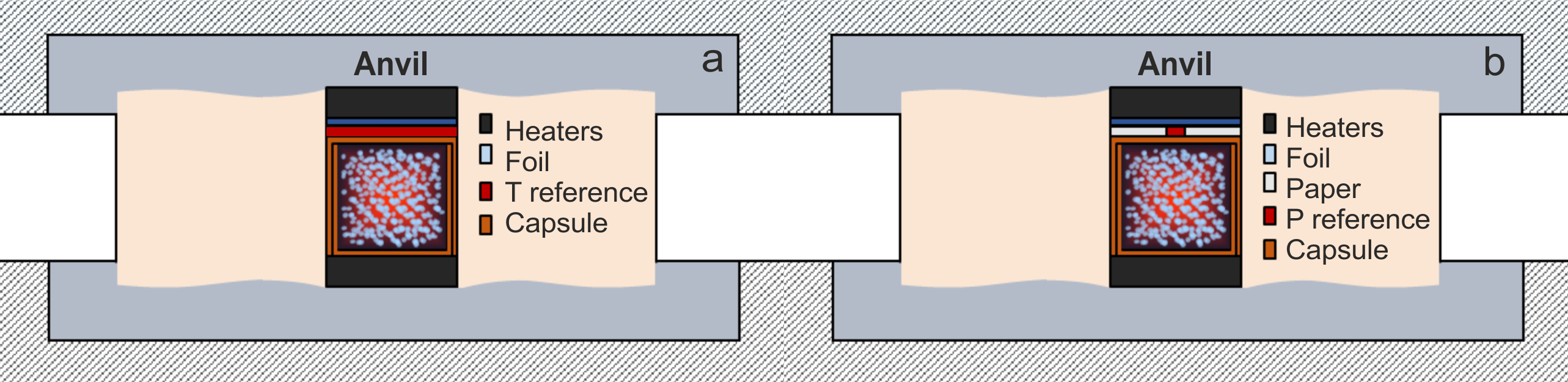


**Supplementary Figure 3**. The schematic Toroid-type high-pressure chamber calibration assemblage: a – temperature calibration; b – pressure calibration. The experimental assemblage includes only the capsule and heaters.


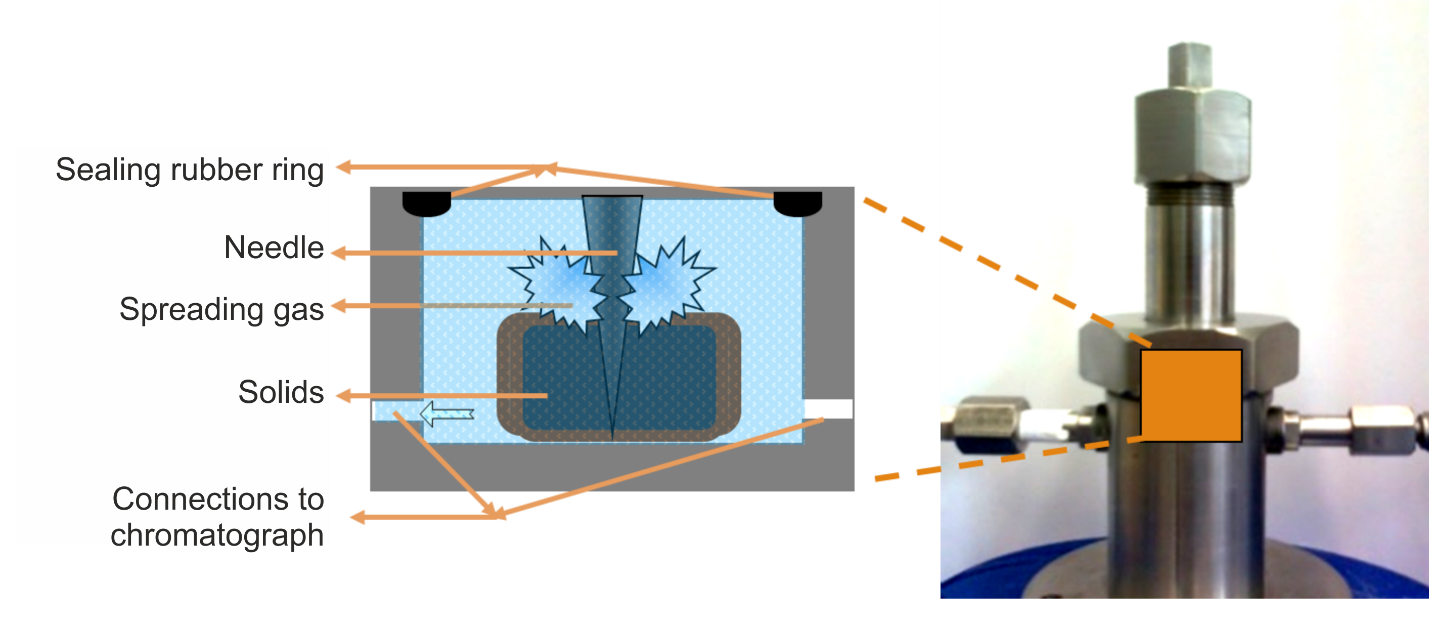


**Supplementary Figure 4.** The gas-extracting cell appliance.

**Supplementary Note**.

The analysis of the hydrocarbon gases produced was performed using gas chromatography. The relative yields of the gases produced were estimated based on the intensity of chromatographic peaks. The experiments conducted at lower temperatures (#7-11) produced minor quantity of hydrocarbons – 10-15 mV of methane from FID detector. The experiments conducted at higher temperatures (#1-6) produced much more hydrocarbons – 500-3000 mV of methane from FID detector.
